# Supplementary material for: WordCommentsAnalyzer: A windows software tool for qualitative research
Source: F1000Res. 2018 Sep 4;7:536. Originally published 2018 May 3. [Version 2] doi: 10.12688/f1000research.14819.2 (PMC6137411; doi:10.12688/f1000research.14819.2)
Supplement: Supplementary file 3 [file f1000research-7-17614-s0002.tgz › b2a79496-4ada-47c4-9682-a99ce867836d.zip!/Eight Iranian Health Journals/Journal of Knowledge and Health_18.docx]

© Journal of Knowledge and Health. This work is licensed under CC BY 4.0 (https://creativecommons.org/licenses/by/4.0/).

Volume & Issue: دوره 8، شماره 4: 1392

Title: بررسي تأثير فعاليت ورزشي تناوبي بر ميزان حجم تومور در موش‌هاي مبتلا به سرطان سينه

http://knh.shmu.ac.ir/index.php/site/article/view/42

مقدمه: فعاليت ورزشي نقش مؤثري در تغيير عوامل مؤثر در سرطان دارد، اما تأثیر فعالیت بدنی بر سطوح عوامل مؤثر سرطان در انسان یا حیوانات مبتلا به آن، تا به‌حال مورد توجه جدی قرار نگرفته است. پژوهش حاضر به بررسي تأثير فعاليت ورزشي استقامتي پيوسته بر ميزان حجم تومور در موش‌هاي مبتلا‌ به سرطان سينه مي‌پردازد. مواد و روش‌ها: در اين مطالعه تعداد 30 سر موش بالب سی ماده (4 تا 5 هفته‌ای با میانگین وزنی 76/17 گرم) خريداري و در ناحيه زيرجلدي شكم آنها تومور آدينوكارسينوماي موشي پيوند زده شد و به‌طور تصادفي به دو گروه تومور- شاهد، تومور- تمرين تناوبي، تقسيم شدند. شدت تمرين در اين پروتكل در هفته اول 20% حداكثر اكسيژن مصرفي و در هفته آخر به 75% حداكثر اكسيژن مصرفي رسيد. بعد از اتمام تمرينات و جداسازي طحال موش، با تست اليزا سنجش پروتئين شوك حرارتي (HSP70)، اينترلوكين 4 (IL4) و اينترفرون گاما (IFNγ) انجام گرفت. نتايج: نتايج نشان داد ميزان HSP70 در گروه تمرين تناوبي (مداخله) نسبت‌به گروه شاهد كمتر است اما اين اختلاف معنادار نبود (05/0P>). همچنين ميزان IL4 در گروه تمرين تناوبي نسبت‌به گروه شاهد كمتر بود، اما معنادار نبود. ميزان IFNγ در گروه تمرين تناوبي نسبت‌به گروه شاهد بالاتر بود اما اين شاخص هم در دو گروه اختلاف معناداري نداشت. درخصوص حجم تومور، ميزان آن در گروه تمرين تناوبي به‌طور معناداري نسبت‌به گروه شاهد كمتر بود (001/0=P). نتيجه‌گيري: طبق يافته‌هاي پژوهش مي‌توان نتيجه گرفت كه فعاليت ورزشي تناوبي با تغيير در ميزان عوامل مؤثر در سرطان مي‌تواند سبب كاهش حجم تومور گردد.

Title: تأثير گیاه سالوادورا پرشيكا بر فرآیند التیام زخم جلدی: مطالعه تجربی در مدل حیوانی

http://knh.shmu.ac.ir/index.php/site/article/view/44

مقدمه: هدف اين مطالعه، بررسی عصاره گیاه سالوادورا پرشيكا در ترمیم زخم جلدی در یک مدل حیوانی مي‌باشد. مواد و روش‌ها: یک زخم جلدی در ناحیه کمر 80 موش نر صحرایی بالغ که به‌طور تصادفی به دو گروه تقسیم شده بودند، ایجاد و روزانه یک بار، سطح زخم گروه شاهد با کرم دارونما و گروه مداخله با پایه کرم حاوی عصاره گیاه سالوادورا پرشيكا پوشانده شد. زخم‌ها در روز 1 از نظر ارتشاح نوتروفیل، در روز 3 از نظر ارتشاح ماکروفاژها و لنفوسیت‌ها، در روز 5 از نظر ارتشاح لنفوست‌ها، فیبروبلاست‌ها، تعداد عروق جدید و بهبودی، در روز 14 از نظر تجمع کلاژن، بهبودی و اندازه زخم و بالاخره در روز 21 از نظر بهبودی و اسکار بررسی شدند. نتايج: میانگین نمره اسکار (± انحراف معيار) در روز 21 در گروه شاهد 78/2±13/8 و در گروه مداخله 79/1±04/3 بود (001/0P<). در روز 14، میانگین نمره بهبودی زخم به‌ترتیب 67/0±76/0 و 48/0±65/1 بود (02/0P=). همچنین میانگین نمره تجمع کلاژن در دو گروه 5/0±6/1 و 34/0±7/2 (002/0P=) و قطر زخم 34/0±2/1 و 23/0±25/0 میلی‌متر گزارش شد (001/0P=). ميزان ارتشاح، تعداد عروق جدید و نمره بهبودی زخم در روزهای 5 و 21 پس از ایجاد زخم، در دو گروه تفاوت معناداری نداشتند. نتیجه‌گیری: مقایسه دو گروه در روزهای 14 و 21 وضعیت کاملاً بهتری را برای گروه مداخله ازنظر تجمع کلاژن، اندازه زخم و نمره اسکار نشان داد. همچنین نمره بهبودی در گروه مداخله در روز 14 بیشتر بود. به‌نظر می‌رسد عصاره گياهي مورد مطالعه بر روند التیام اثر مطلوب داشته باشد.

Title: بررسی شیوع وابستگی به موبایل و ارتباط آن با عزت‌نفس دانشجویان

http://knh.shmu.ac.ir/index.php/site/article/view/46

مقدمه: وابستگی به تلفن همراه مشکلی جدی برای کار و زندگی اجتماعی است. افرادی با عزت‌نفس پایین، استفاده‌ی مشکل‌ساز و بیشتری از تلفن همراه دارند. هدف مطالعه تعيين شيوع وابستگي به موبايل و ارتباط آن با عزت‌نفس دانشجويان است. مواد و روش‌ها: در این مطالعه توصیفی- تحلیلی 697 دانشجو با روش نمونه‌گیری تصادفی سیستماتیک انتخاب شدند. ابزار جمع‌آوري داده‌ها پرسشنامه‌ي عزت‌نفس 10 سؤالی روزنبرگ با مقياس 4 درجه‌اي ليكرت و پرسشنامه وابستگی به موبایل شامل 20 سؤال در سه حیطه: تحمل محرومیت (سؤالات 1-7)، اختلال عملکرد زندگی (سؤالات 8-13) و اجبار- اصرار (سؤالات 14-20) بود. داده‌ها در نرم‌افزار SPSS وارد و با آزمون‌های آماری t-test و کای- دو و ضریب همبستگی پیرسون آنالیز شدند. نتایج: ميزان شيوع وابستگي به موبايل 9/0% مي‌باشد. ميزان وابستگي به موبايل در دو جنس تفاوت معناداري نداشت، اما برحسب سن، رشته، مقطع تحصیلی، مدت و چگونگی استفاده، تعداد تماس و پیام‌های دریافتی و ارسالی روزانه و متوسط شارژ ماهانه متفاوت بود. مدت استفاده از موبایل و تعداد تماس و پیام‌های دریافتی و ارسالی روزانه و ميزان استفاده از اينترنت موبایل و متوسط شارژ ماهانه در دانشجویان برحسب سن و جنس و رشته و مقطع تحصیلی به‌طور معناداري متفاوت بود. بين وابستگي به موبايل و عزت‌نفس در دانشجويان همبستگي معنادار منفي وجود داشت (67/0-=r). نتیجه‌گیری: کاهش عزت‌نفس می‌تواند منجر‌به استفاده مشکل‌ساز از تلفن همراه شود. پس شناسايي افراد در معرض خطر و به کار گرفتن راه‌کار‌هايي براي ارتقای عزت‌نفس مفید مي‌باشد.

Title: نگرش پزشکان در به‌کارگیری پزشکی مبتنی‌بر شواهد و موانع مربوط‌به آن

http://knh.shmu.ac.ir/index.php/site/article/view/47

مقدمه: پزشکی مبتنی‌بر شواهد استفاده درست، صحیح و خردمندانه از بهترین شواهد در تصمیم‌گیری بالینی برای مراقبت از بیماران است. مطالعات نشان داده‌اند، استفاده از شواهد معتبر در مراقبت‌هاي باليني تحت تأثير نگرش افراد قرار دارد. هدف این مطالعه بررسی نگرش پزشکان و موانع به‌کارگیری پزشکی مبتني‌بر شواهد در کشور ایران است. مواد و روش‌ها: اين پژوهش از نوع توصیفی- مقطعی است. جامعه پژوهش، پزشکان عمومی، رزیدنت‌ها و متخصصین بیمارستان‌های کشور ایران در سال 1390 بودند. حجم نمونه 245 نفر تعیین شدکه به‌صورت تصادفی ساده از بین جامعه پژوهش انتخاب گردید. جمع‌آوری داده‌ها با استفاده از پرسشنامه، مشتمل بر اطلاعات زمینه‌ای و اطلاعات مبتنی‌بر اهداف پژوهش صورت پذیرفت. روايي صوری و محتوی با تأیید افراد صاحب نظر و پايايي آن 8/0=α به‌دست آمد. نتایج: در رابطه با نگرش، 3/85% پزشکان موافق بودند که پزشکی مبتنی‌بر شواهد، مراقبت بیماران را بهبود می‌بخشد، 8/71% این رویکرد را در امر تصمیم‌گیری باليني کمک‌کننده ذكر كردند و 89% نیز بهره‌مندی پزشکان از کتابخانه دیجیتال را در به‌کارگیری پزشکی مبتنی‌بر شواهد موثر اعلام نمودند. 2/59% عدم‌سرمایه‌گذاری و فقدان تشویق مادی و معنوی برای پزشکان و 3/56% نیاز به آموختن مهارت‌های جدید بسیار را، بزرگترین مانع در به‌کارگیری آن عنوان کردند. نتیجه‌گیری: با آنکه پزشکان نگرش موافقی نسبت‌به پزشکی مبتنی‌بر شواهد دارند؛ عدم‌سرمایه‌گذاری و فقدان تشویق‌های مادی و معنوی، موجب بی‌توجهی پزشکان در به‌کارگیری پزشکی مبتنی‌بر شواهد در طبابت شده است.

Title: بررسی اثر تمرینات تصویرسازی ذهنی بر تغییرات فعالیت الکتریکی واحدهای حركتي عضلات و قدرت آنها در اندام تحتانی

http://knh.shmu.ac.ir/index.php/site/article/view/49

مقدمه: هدف از اجرای این تحقیق بررسی اثر تمرینات تصویرسازی ذهنی بر تغییرات فعالیت الکتریکی واحدهای حركتي عضلات و قدرت آنها در اندام تحتانی می‌باشد. مواد و روش‌ها: در اين مطالعه 30 دانشجوي تمرین نکرده، با میانگین سنی 2/1±4/22 سال، قد 6/5± 2/176 سانتي‌متر و وزن 2/6±6/67 كيلوگرم شرکت داشته كه به‌طور تصادفي به دو گروه تمرين ذهني و شاهد تقسيم شدند. گروه تجربی، انقباضات بیشینه ارادی عضلات پلانتار فلکسور مچ پا را پنج جلسه در هفته و به‌مدت چهار هفته تصویرسازی کردند. گروه شاهد در هیچ فعالیت جسماني یا برنامه تمرین ذهنی شرکت نداشتند. برنامه تمرین شامل تصویرسازی 50 انقباض بیشنه ارادی در 2 نوبت 25 تکراری بود. برای تجزیه‌و‌تحلیل داده‌ها از آزمون t در سطح معناداری 05/0P≤ استفاده شد. نتايج: نتايج نشان داد كه بين اندازه‌گيري‌هاي انتگرال الكتروميوگرافي عضله دوقلو مربوط‌به گروه شاهد و تجربي در پس آزمون تفاوت معناداري وجود دارد و در گروه تجربي افزايش معناداري در پارامترهاي موردنظر نسبت‌به گروه شاهد رخ داد. همچنین بين اندازه‌گيري‌هاي انتگرال الكتروميوگرافي عضله ساقي قدامي مربوط‌به گروه شاهد و تجربي در پس آزمون تفاوت معناداري وجود دارد. این افزایش نیرو به تغییرات ایجاد شده در برنامه‌ریزی مرکزی سیستم عصبی نسبت داده مي‌شود که موجب افزایش سطح فعال‌سازی عضلات موافق وکاهش آن در عضلات مخالف حین عمل عضلات موافق شده است. نتیجه‌گیری: نتایج نشان داد تمرینات تصویرسازی ذهنی می تواند باعث افزایش قدرت عضلات و فعالیت الکتریکی واحدهای حرکتی عضلات گردد.

Title: مطالعه کیفیت زندگی دانشجویان در یکی از دانشگاه‌های علوم پزشکی شمال‌شرق ایران

http://knh.shmu.ac.ir/index.php/site/article/view/54

مقدمه: کیفیت زندگی به‌عنوان شاخص مهمی برای ارزیابی سلامت فردی، تصمیم‌گیری و قضاوت در مورد سلامت کلی جامعه و یافتن مشکلات اصلی در ابعاد گوناگون زندگی افراد است. مطالعه حاضر با هدف تعیین کیفیت زندگی دانشجویان دانشگاه علوم پزشکی شاهرود انجام شده است. مواد و روش‌ها: اين پژوهش به شیوه مقطعي در سال 1390 انجام شد. در اين مطالعه از پرسشنامه 26 سؤالی کیفیت زندگیWHO  استفاده گردید. داده‌های به‌دست آمده با استفاده از آزمون کای- ‌دو به کمک نرم‌افزار آماریSPSS  مورد تجزیه‌و‌تحلیل قرار گرفتند. نتايج: میانگین سنی افراد مورد مطالعه 3/2±8/20 سال بود. 2/79% از افراد را دختران تشکیل می‌دادند. 7/22% دانشجویان بومی بودند. ميانگين نمره کیفیت زندگی دانشجویان در حیطه‌های 4 گانه سلامتی شامل بُعد سلامت جسمانی (4/2±2/14)، سلامت اجتماعی (2/3±1/14)، سلامت روانی (8/2±1/13) و سلامت محیطی (5/2±7/12) بود. رابطه معناداری بین کیفیت زندگی و جنس، مقطع تحصیلی، ترم تحصیلی، وضعیت تأهل، محل سکونت، فعالیت اقتصادی، تعداد افراد خانواده، رتبه تولد، در قید حیات‌بودن والدین مشاهده نگردید (05/0<P). ولی ارتباط معناداری بین کیفیت زندگی با بومی‌بودن (049/0P=)، درآمد خانواده (02/0P=) و رشته تحصیلی (02/0P=) وجود داشت. نتيجه‌گيري: علاوه‌بر بومی‌گزینی دانشجویان در سطح دانشگاه‌ها، راه‌اندازی کلینیک‌های مشاوره در محیط دانشگاه و خوابگاه، برگزاری دوره‌های توجیهی و آموزشی درخصوص شیوه‌های زندگی در محیط‌های دانشجویی، افزایش امکانات و برنامه‌های ورزشی و تفریحی، می‌تواند سبب ارتقای سطح سلامتی دانشجویان و بهبود کیفیت زندگی آنان گردد.

Title: اثر بربرين ‌هيدروكلرايد بر اختلالات عملکرد حرکتي در موش‌هاي صحرايي ديابتي‌شده با استرپتوزوتوسين

http://knh.shmu.ac.ir/index.php/site/article/view/55

مقدمه: بيماري ديابت منجر‌به بروز اختلالات حرکتي گرديده و بربرين هيدروكلرايد، آلكالوئيدي با آثار متعدد ضد ديابتي و آنتي‌اكسيدان مي‌باشد. اين مطالعه با هدف بررسي تأثير بربرين هيدروکلرايد بر اختلالات حرکتي ناشي از ديابت در موش‌هاي صحرايي ديابتي‌شده با استرپتوزوتوسين طراحي شده است. مواد و روش‌ها: در اين مطالعه، 40 سر موش صحرايي نر نژاد ويستار انتخاب و به‌طور تصادفي به پنج گروه مساوي: شاهد، شاهد تيمارشده با بربرين هيدروكلرايد (mg/kg100)، ديابتي و ديابتي تيمارشده با بربرين‌هيدروكلرايد (mg/kg100 و 50) تقسيم شدند. ديابت با تزريق استرپتوزوتوسين با دوز 55 ميلي‌گرم/ كيلوگرم به‌صورت درون صفاقي القاء گرديد. يك هفته پس از تزريق استرپتوزوتوسين، تيمار با بربرين هيدروكلرايد با دوز 50 و 100 ميلي‌گرم/روز به‌مدت شش هفته به‌صورت خوراكي انجام شد. اندازه‌گيري قندخون نيز در هفته‌هاي 1، 3، 5 و 7 ازطريق سياهرگ دمي به‌عمل آمد و در پايان هفته ششم و هفتم، گروه‌ها با آزمون‌هاي رفتاري، سطح شيب‌دار، بارفيکس و ماز y مورد ارزيابي قرارگرفتند. نتايج: گروه ديابتي تيمارشده با بربرين هيدروكلرايد (mg/kg100 و 50) بهبودي قابل‌توجهي را در مقايسه با گروه ديابتي در آزمون‌هاي سطح شيب‌دار، بارفيکس و ماز y نشان دادند. نتيجه‌گيري: تجويز بربرين هيدروكلرايد به‌مدت شش هفته منجربه بهبود اختلالات حرکتي در موش‌هاي صحرايي ديابتي‌شده با استرپتوزوتوسين مي‌شود.

Title: بررسی سیستم نمره‌دهی آپاچي- دو در پیش‌بینی نتایج جداسازی از ونتیلاتور

http://knh.shmu.ac.ir/index.php/site/article/view/433

مقدمه: ظهور سیستم‌های نمره‌دهی آی‌ سی‌ یو (ICU) در راستای بهبود نتایج بخش‌های مراقبت ویژه ازطریق پیش‌بینی نتایج، گام مؤثری در جهت جداسازی سریع و به‌موقع بیماران از ونتیلاتور بوده است. در این مطالعه ارتباط بین نمره آپاچی- دو و سرانجام بيماران (نجات‌يافته، فوت‌شده) بستري مورد بررسی قرار گرفته است. مواد و روش‌ها: در این مطالعه 300 بیمار بستری در آی‌سی‌یو جنرال (بيمارستان امام علي(ع) بجنورد) و آی‌سی‌یو جراحی (بيمارستان فارابي مشهد) و در یک دوره 6 ماهه مورد بررسي قرار گرفتند. جمع‌آوری داده‌هاي مربوط‌به انتخاب نمونه و مشخصات فردی با استفاده از پرسشنامه و داده‌هاي مربوط‌به فرم آپاچي- دو به روش ثبت از پرونده صورت گرفت. ورود داده‌های مربوط‌به فرم آپاچی- دو ناوس (1985) که شامل سه قسمت: نمره فیزیولوژیک حاد، نمره تطبیق بیماری‌های مزمن زمینه‌ای و نمره تطبیق سن می‌باشد، در 24 ساعت اول پذیرش با رعایت معیار حذف و شمول، انجام شد. نتايج: بين ميانگين نمره آپاچي= دو 24 ساعت اول پذيرش برحسب سرانجام بيماري در دو بيمارستان تحت مطالعه تفاوت معناداري مشاهده شد. همچنين بين سطوح كراتينين سرم در دو گروه نجات‌يافته و فوت‌شده اختلاف آماري معناداري مشاهده شد (003/0=P). افزایش نمره آپاچی- دو 24 ساعت اول پذیرش در هر دو بیمارستان با افزایش مرگ‌و‌میر بیماران ارتباط آماری معناداری نشان داد. تمام بیماران با نمره آپاچی- دو بالاتر از 24 فوت نمودند و تمامی بیماران با نمره آپاچی- دو کمتر از 10 زنده ماندند. میزان مرگ‌و‌میر بیماران تحت مطالعه 23% بود. نتیجه‌گیری: نتايج این مطالعه نشان داد که بين نمره آپاچي- دو 24 ساعت اول پذيرش با سرانجام بيماران (فوت شده و نجات‌يافته) رابطه معناداري وجود دارد به‌صورتي‌كه افراد با نمره آپاچي- دو بالاتر، ميزان مرگ‌و‌مير بيشتري نسبت‌به افراد با نمره آپاچي- دو كمتر داشتند.

Title: شيوع و پیامد‌های ناامني غذایی و ارتباط آن با برخی عوامل اجتماعی- اقتصادی

http://knh.shmu.ac.ir/index.php/site/article/view/388

مقدمه: ناامني غذایی، مي‌تواند بر سلامت جسمي و رواني افراد تأثيرگذار باشد. هدف از این مطالعه بررسی وضعیت ناامنی غذایی و برخی عوامل اجتماعی- اقتصادی مرتبط با آن، در خانواده‌های دانش‌آموزان دختر مقطع ابتدایی شهرستان بندر انزلی، واقع در شمال ایران مي‌باشد. مواد و روش‌ها: اين مطالعه مقطعی با هدف بررسي وضعیت ناامني غذایی در خانوارهای 400 دانش‌آموز دختر 9 تا 11 ساله انجام شده است. جهت تعیین برخی ویژگی‌های دموگرافیک و فردی خانوار و تعیین میزان ناامني غذایی، مادران دعوت شدند و پرسش نامه‌های اطلاعات اجتماعی- اقتصادی و پرسشنامه 18 سؤالی وضعیت امنیت غذایی سازمان كشاورزي ايالات متحده (US Department of Agriculture) طی مصاحبه حضوری تكميل شدند. براي تعيين وضعيت اقتصادي از وجود 9 قلم از وسايل زندگي سؤال شد. داده‌ها با استفاده از روش آناليز واريانس يك‌طرفه و رگرسيون لجستيك چند متغيره تجزيه‌وتحليل شدند. نتايج: میزان شیوع ناامني غذایی در جامعه مورد مطالعه 51% بود. ناامني غذایی با سطح اقتصادی خانواده، تعداد افراد شاغل خانواده، تعداد فرزندان خانواده، سطح تحصیلات پدر و مادر و مالکیت منزل ارتباط معناداری داشت. در گروه خانواده‌هاي با ناامني غذايي، ميانگين وزن كودك به‌طور معناداري كمتر از گروه ديگر بود. نتيجه‌گيري: ناامني غذایی به شدت تحت تأثیر سطح اقتصادی، تحصیلات و جایگاه شغلی والدین مي‌باشد.

Title: پارگي دئودنوم متعاقب تروماي غيرنافذ شكم

http://knh.shmu.ac.ir/index.php/site/article/view/57

پارگي دئودنوم به‌دنبال ترومای غیرنافذ (Blunt) معمولاً خيلي نادر بوده و به‌دنبال ضربه به ناحیه اپي‌گاستر متعاقب تروما، تصادف و يا آسيب‌هاي ورزشي رخ مي‌دهد. به‌دليل عدم‌وجود علائم اختصاصي، معمولاً تشخيص با تأخير صورت گرفته و عوارض خطرناك و گاه كشنده‌اي را به همراه خواهد داشت. بيمار موردنظر آقاي 29 ساله‌اي است كه 3 روز بعد از تصادف با اتومبیل با درد شديد شكم، تهوع، كاهش سطح هوشياري و بی‌ثباتی علایم حیاتی به اورژانس بيمارستان امام حسين(ع) شاهرود مراجعه نموده است. پس از انجام اقدامات اوليه و تثبیت علایم حیاتی بيمار با تشخيص خونریزی داخلی و پريتونيت به‌طور اورژانسی تحت عمل جراحی لاپاراتومي قرار گرفت كه پارگي عرضي و وسیع قسمت سوم دئودنوم مشخص گردید. بعد از ترميم دئودنوم، درن در محل عمل تعبیه و بيمار در ICU بستري و داروهای لازم تجویز شد. دو روز بعد از عمل، به‌دنبال ليك از محل درن شكمي، مجدداً لاپاراتومي و دئودنوستومي ترمیم شد. یک هفته بعد مجددأ به‌دلیل لیک درن، بیمار تحت لاپاراتومي قرار گرفته و ژژونوستومي براون و دئودنوستومي مجدد تعبیه شد. يك هفته بعد از آخرين عمل، با بهبود وضع عمومي و شروع تغذيه از راه ژژونوستومی درن‌ها خارج و بیمار با حال عمومی خوب ودستورات دارویی مرخص شد. پس می‌توان با سرعت عمل كافي، دقت در علائم و نشانه‌هاي باليني و معاينه دقيق بيماران فوق، ضمن جلوگيري از اتلاف وقت و هزينه، نتايج درماني مطلوبی را به‌دست آورد.
